# Unraveling the Thermodynamics of Ultra-tight Binding of Intrinsically Disordered Proteins

**DOI:** 10.3389/fmolb.2021.726824

**Published:** 2021-08-31

**Authors:** Uroš Zavrtanik, San Hadži, Jurij Lah

**Affiliations:** Department of Physical Chemistry, Faculty of Chemistry and Chemical Technology, University of Ljubljana, Ljubljana, Slovenia

**Keywords:** intrinsically disordered proteins, ultra-high affinity, picomolar, protein-protein interactions, conditional folding, fuzzy interactions, toxin-antitoxin

## Abstract

Protein interactions mediated by the intrinsically disordered proteins (IDPs) are generally associated with lower affinities compared to those between globular proteins. Here, we characterize the association between the intrinsically disordered HigA2 antitoxin and its globular target HigB2 toxin from *Vibrio cholerae* using competition ITC experiments. We demonstrate that this interaction reaches one of the highest affinities reported for IDP-target systems (*K*_D_ = 3 pM) and can be entirely attributed to a short, 20-residue-long interaction motif that folds into α-helix upon binding. We perform an experimentally based decomposition of the IDP-target association parameters into folding and binding contributions, which allows a direct comparison of the binding contribution with those from globular ultra-high affinity binders. We find that the HigA2-HigB2 interface is energy optimized to a similar extent as the interfaces of globular ultra-high affinity complexes, such as barnase-barstar. Evaluation of other ultra-high affinity IDP-target systems shows that a strategy based on entropy optimization can also achieve comparably high, picomolar affinities. Taken together, these examples show how IDP-target interactions achieve picomolar affinities either through enthalpy optimization (HigA2-HigB2), resembling the ultra-high affinity binding of globular proteins, or via bound-state fuzziness and entropy optimization (CcdA-CcdB, histone H1-prothymosin α).

## Introduction

In order to perform their biological function proteins interact with their partners and establish a complex network of protein-protein interactions. Understanding the structural and thermodynamic properties of protein-protein interactions has historically focused on proteins with a well-defined three-dimensional structure, namely globular proteins (Jones and Thornton, 1996; Conte et al., 1999). In many cases, interactions between such well-folded proteins can be viewed through the classical paradigm of the lock-and-key mechanism, where the interaction surfaces are highly complementary and no major conformational changes are observed upon binding (Chothia and Janin, 1975). Still, some conformational changes can be observed upon binding of globular proteins, which have important implications for their function. The most extreme examples of conformational changes associated with protein binding can be found among the intrinsically disordered proteins (IDPs), which do not have a compact three-dimensional structure in their native state. Upon binding to target proteins IDPs become structured to varying degrees ranging from completely ordered complexes, usually involving formation of α-helices, to those which retain a high degree of disorder in the bound state, so-called fuzzy complexes (Sugase et al., 2007; Tompa and Fuxreiter, 2008; Fuxreiter, 2019).

Physiological affinities of protein-protein interactions span several orders of magnitude from milli to femtomolar range. At the high affinity end the strongest characterized associations between globular proteins reach femtomolar affinities and have been observed for various types of ribonucleases and their cognate inhibitors, such as barnase-barstar, and complexes between bacterial colicins (E-colicins) and immunity proteins (Im) (Hartley, 1989; Kleanthous et al., 1998). On the other hand, it has been previously postulated that IDPs cannot form such high affinity complexes because they need to fold upon binding, which is accompanied by a significant entropic penalty that lowers the association affinity (Dunker et al., 2001). Along the same line it has been hypothesized that IDPs employ various structural and thermodynamic strategies to reduce this entropic penalty, for example by pre-folding or by retaining a high degree of dynamics in the bound state (Pancsa and Fuxreiter, 2012; Flock et al., 2014). The large-scale comparison of the free energies of protein-protein associations has shown that associations involving IDPs are on average 2 to 2.5 kcal/mol less favorable than those between a pair of globular proteins (Huang and Liu, 2013; Teilum et al., 2015). A recent survey complexes involving globular proteins and those involving IDPs also observed that the interaction surfaces involving one IDP partner are more frustrated and less energy optimized (Freiberger et al., 2021; Gianni et al., 2021). Unfortunately, a direct comparison of thermodynamic profiles between IDP-protein and globular protein-protein association is not meaningful, since the thermodynamic profile of IDP-protein interactions is strongly affected by the contributions related to IDP conformational changes. In particular a comparison of the enthalpic contribution to the overall association free energy would be informative, as it reflects the establishment of interactions upon binding (Ladbury et al., 2010). However, for the IDP-target associations the measured enthalpies contain an additional contribution related to the IDP folding, since formation of helices is accompanied with a considerable enthalpy change (Richardson et al., 2005).

**FIGURE 1:**
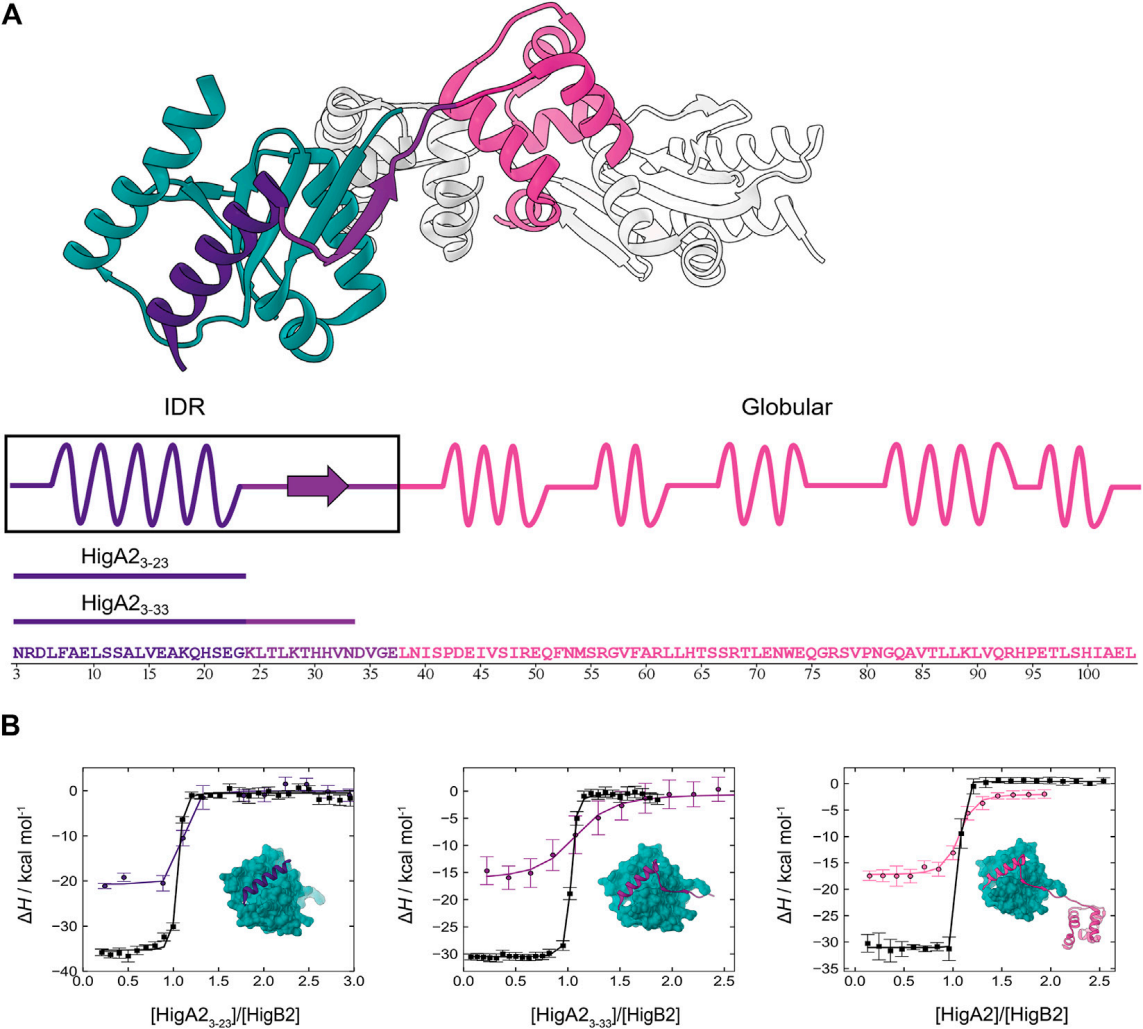
Intrinsically disordered HigA2 binds to its globular target with picomolar affinity. **(A)** The overall structure of HigA2-HigB2 antitoxin-toxin tetramer (PDB 5JAA). Only one pair of chains (heterodimer) is colored for clarity. Globular toxin HigB2 is shown in dark cyan, while three antitoxin's structural segments are shown in indigo (IDP α-helix motif), violet (IDP β-strand motif) and pink (globular domain). Underneath the sequence of HigA2 antitoxin and its truncated versions is shown in the same color scheme. **(B)** The ITC binding isotherms corresponding to titrations of different HigA2 segments into HigB2 (direct titration in black) or into the nanobody-HigB2 complex (competition titration in indigo, violet and pink). The inset shows the expected final product of titrations and the lines correspond to global fit of direct and competition titrations.

In this study, we investigated the interaction between the intrinsically disordered bacterial antitoxin HigA2 and its globular target HigB2 from *Vibrio cholerae* and found that the association is characterized by an unprecedentedly high, picomolar affinity *K*_D_ = 3 pM. Previous structural studies identified two domains in the HigA2 antitoxin: the intrinsically disordered N-terminal part (residues 1–37) and the fully structured C-terminal helix-turn-helix dimerization and a DNA-binding domain (residues 38–105) (Hadži et al., 2017a). The crystal structure of HigA2-HigB2 complex shows that the antitoxin wraps around the toxin with its N-terminal intrinsically disordered domain and forms an elongated heterotetrameric structure (Figure 1A). Upon binding the disordered domain folds into an α-helix (residues 3–22) followed by a short β-strand (residues 28–32) that expands the existing β-sheet core of HigB2 toxin (Figure 1A). Most of the interactions with toxin are mediated through the N-terminal part of HigA2 (the α-helix and the β-strand), however some additional contacts are also established by the globular part of the antitoxin. We performed a series of competition ITC experiments to elucidate the thermodynamic profile of ultra-tight HigA2-HigB2 association. Using the theoretical framework of the helix-coil transition theory, we estimated the contribution related to HigA2 folding and used it to decompose the overall association thermodynamics into folding and binding contributions. The obtained HigA2-HigB2 binding contribution (independent of IDP folding effects) could now be compared to those from the globular ultra-high affinity binders. We observed that the HigA2-HigB2 interactions are optimized in terms of potential energy (enthalpy) to a similar extent as those in the interfaces of globular high affinity binders. Interestingly, a comparison with other ultra-tight IDP-target complexes such as CcdA-CcdB and histone H1- prothymosin α (ProTα) shows that IDP systems employ different degrees of enthalpy and entropy optimization to achieve ultra-high association affinities.

## Results

### Competition ITC Experiments Enable Characterization of the Ultra-high Affinity IDP-Target Association

Previously, we observed that the association between the intrinsically disordered antitoxin HigA2 and its target toxin HigB2 is governed by a very high affinity. However, due to the lack of a suitable competitive ligand the previous estimate of the HigA2-HigB2 affinity was based on temperature extrapolation of the association free energy which, as we show now, led to a significant underestimation (Hadži et al., 2017a). The ITC isotherms accompanying binding of the full-length HigA2 to HigB2 show a very sharp, step-like transition (Figure 1B, black line on the far-right panel) that precludes a reliable estimate of affinity from a direct ITC titration. We next tested whether nanobodies raised against the toxin HigB2 could be used as binding competitors. We found that the nanobodies Nb6 (binding to the C-terminal helix of HigB2 (Hadži et al., 2017a)) and Nb10 compete with the HigA2 binding and performed the competition ITC experiments by titrating HigA2 into the preformed nanobody-HigB2 complex (see *Materials and Methods*). Although the nanobodies alone bind to the toxin HigB2 with a relatively high affinity (Table 1, Supplementary Figure S1), the resulting competition isotherms are still relatively steep, already indicating a very tight HigA2-HigB2 binding (Figure 1B, colored lines). Global fitting of the direct and the competition ITC isotherms leads to the accurate estimation of the HigA2-HigB2 affinity, which is *K*_D_ = 16 pM at 25°C (Δ*G*_assoc_ = −14.7 kcal mol^−1^). The association of HigA2-HigB2 is driven by a strongly favorable enthalpy change (Δ*H*_assoc_ = −31.5 kcal mol^−1^) and counteracted by the unfavorable entropy change (−TΔ*S*_assoc_ = 16.8 kcal mol^−1^). Together with the previously measured temperature-dependent ITC data used to estimate the heat capacity contribution (Hadži et al., 2017a) this provides us with a complete thermodynamic profile accompanying the association of HigA2 with its target HigB2 (Table 1).

**TABLE 1:** Thermodynamics of HigA2 and HigB2 association derived from the ITC experiments. Parameters are reported at T = 25°C.

Association		Δ*G*/kcal mol^−1^	Δ*H*/kcal mol^−1^	−TΔ*S/*kcal mol^−1^	Δ*C*_p_/kcal mol^−1^ K^-1^
HigB2 +	Full-length HigA2	−14.7 ± 0.9	−31.5 ± 1.0	16.8 ± 1.9	−0.95^a^
	HigA2_3-33_	−13.5 ± 1.9	−30.3 ± 1.0	16.8 ± 2.9	
	HigA2_3-23_	−15.7 ± 1.9	−34.6 ± 1.0	19.1 ± 2.9	−0.46 ± 0.02
	Nb10	−10.4 ± 0.2	−16.2 ± 0.6	5.8 ± 0.8	
	Nb6	−10.9 ± 0.2	−14.2 ± 0.4	3.3 ± 0.6	

a (Hadži et al., 2017a).

### The α-helical Motif Is the “Hot Region” Contributing Most of the Association Free Energy

To explore the origins of the high affinity HigA2-HigB2 association, we designed several truncated variants of HigA2 covering different segments of the intrinsically disordered region (Figure 1A). The longer peptide HigA2_3-33_, comprises the segment that folds into an α-helix and a β-strand upon binding, corresponding to residues 3–33. The shorter peptide HigA2_3–23_ covers only a segment that forms an α-helix upon binding to the toxin, corresponding to residues 3–23. As described above for the full-length HigA2, we performed the ITC competition experiments using the HigA2 peptides and the nanobodies Nb6 or Nb10 as competitors. Figure 1B shows the binding isotherms for direct (black lines) and competition titrations (colored lines) for both peptide variants. Overall, the isotherms for all three binders (HigA2_3–23_, HigA2_3–33_ and HigA2) look similar and the resulting thermodynamic parameters are not significantly different between these three systems (Table 1). This means that only a small segment of the HigA2, namely the α-helix-forming segment HigA2_3–23_, is responsible for virtually all of the association free energy. Thus, it appears that the interactions mediated by the addition of the β-strand or the globular domain of HigA2 do not contribute significantly to the HigA2-HigB2 affinity. In fact, the affinity of HigA2_3-23_ alone *K*_D_ = 3 pM is even somewhat higher than that of full-length HigA2 (Table 1). Most strikingly, the association of the disordered HigA2_3–23_ α-helical motif has, to our knowledge, one of the highest affinities measured to date for IDP-target systems.

Using a computational method we identified the key residues that make dominant contributions to the association free energy, also known as hotspot residues (Sukhwal and Sowdhamini, 2013). In accordance with our experiments that identified the intrinsically disordered α-helix forming segment as a key interaction motif, all the identified hotspot residues (Arg 4, Leu 6, Phe 7, Leu 10, Ala 13 and Leu 14) are located in this segment (Supplementary Figure S2A). These hotspots are mainly hydrophobic and separated by 3 or 4 residues, forming an amphipathic helix. Hydrophobic hotspot residues (Leu 6, Leu 10, Ala 13 and Leu 14) form hydrophobic interaction network and are important for formation of extended hydrophobic core with HigB2’s β-sheet surface. On the other hand, Arg 4 and Phe 7 contribute to the stability of the complex by formation of cation:π interaction with Tyr 14 and Gln 94/97 from HigB2, respectively. Similar results are also obtained from the computational alanine scanning mutagenesis (Wood et al., 2020), which shows that substitutions of these residues to alanine are strongly unfavorable (ΔΔ*G*_Xaa->Ala_ > 7 kcal mol^−1^) and approximately similar in value (Supplementary Figure S2B). In contrast, substitutions of other HigA2 residues are associated with much smaller penalties, except perhaps for Glu 16, which is also located on the α-helix forming segment.

To experimentally verify the significance of those hotspot residues, we designed a variant of the HigA2_3–23_ peptide with several hotspot residues substituted to alanine (HigA2_3–23_ bearing Arg4Ala, Leu6Ala, and Phe7Ala mutations), which presumably eliminates the hotspot-mediated interactions. The peptide with the mutated hotspots completely lost its ability to bind the toxin, as we were unable to detect any signal by ITC titration and did not observe any change in the CD signal upon addition of toxin that would indicate a folding upon binding process (Supplementary Figure S3). This demonstrates the importance of the interactions mediated by the hotspot residues located on the α-helical IDP motif. Overall, these results show that the α-helical interaction motif represents the “hot region” for the interaction with the globular target and is responsible for the ultra-high association affinity.

### Experimental Estimation of the IDP Folding and Binding Contributions

The determined thermodynamic parameters, listed in Table 1, correspond to the overall association process which contains two main contributions: 1) transition of the IDP from the unstructured to the helical conformation (folding) and 2) formation of IDP-target interactions (binding). Clearly, a decomposition into the folding and binding contributions is thermodynamically justified, since the thermodynamic functions considered are state functions and therefore do not depend on the actual kinetic mechanism of coupled binding and folding. Another factor contributing to the overall association thermodynamics involves a shift of HigB2 C-terminal α-helix upon antitoxin binding, as seen in crystal structures of Nb-HigB2 and HigA2-HigB2 complexes (Hadži et al., 2017a). We suspect however that this contribution is not very significant and treat it as being part of the binding contribution. First, the shift of the C-terminal α-helix does not involve any change in the secondary structure (folding/unfolding) and is also not associated with significant changes in the solvent accessible protein surface area. Displacement of the HigB2’s C-terminal helix (free -> bound) leads to the exposure of 112 Å^2^ of apolar and burial of 83 Å^2^ of polar surface area, resulting in the overall change in ASA of only around 30 Å^2^. Furthermore, the NMR characterization of the structural analogue RelE-RelB, has shown that the C-terminal helix exhibits increased mobility and dynamics as evidenced by low hetero NOE and a high transverse relaxation rate (Li et al., 2009). Although there is no NMR data available for HigB2, analysis of the crystallographic B-factors indicates that, similar to RelE, the C-terminal helix is, together with several loops, one of the most flexible parts of the structure. For these reasons the shift of HigB2 C-terminal helix is most likely not associated with a significant energy penalty which is, as explained above, included in the Δ*F* binding term. Next, we investigate the origins of the picomolar affinity observed for the short peptide HigA2_3–23_, which adopts an almost fully folded α-helix upon binding, by decomposing its folding and binding contributions.

The thermodynamic parameters associated with HigA2_3-23_ helical folding can be determined under the framework of helix-coil transition theory by using the Lifson-Roig (LR) model (Lifson and Roig, 1961). The CD spectroscopic measurements of isolated HigA2_3–23_ show that some residual structure, about 10% helicity, is already present in the unbound state (Supplementary Figure S4). The degree of residual helicity is associated with the value of Lifson-Roig helix propagation parameter *w*, which can be determined by fitting the LR homopolymer model to the experimental helicity (Eq. 1, see *Materials and Methods*). The average per-residue helix propensity of HigA2_3-23_ is *w* = 1.1 at 25°C, corresponding to the free energy of 56 cal mol^−1^ for transition of residue from coil to helix. We then estimate the HigA2_3-23_ folding free energy by evaluating the partition function of HigA2_3-23_ in the free state and in the bound state, where we assumed that all residues adopting helical conformation as observed in the X-ray structure are weighted by the obtained parameter *w* (Eq. 2, see *Materials and Methods*). The resulting Gibbs free energy associated with HigA2_3-23_ folding is Δ*G*_fold_ = 2.8 kcal mol^−1^. The positive value of Δ*G*_fold_ indicates that the folding of the peptide into the bound-state conformation is an unfavorable process.

While Δ*G*_fold_ gives us information about the overall folding penalty an additional insight could be gained by evaluating the corresponding enthalpic and entropic folding contributions. To estimate the enthalpy change (Δ*H*_fold_) associated with HigA2_3–23_ folding we use the calorimetric data of Richardson et al. who reported the enthalpic helix-coil contributions (Δ*H*_ch_) for the uncharged amino acids (Richardson et al., 2005). The enthalpic contributions of charged residues can be estimated based on the reasonably good correlation between the consensus helix propensity scale (Pace and Scholtz, 1998) with the Richardson's Δ*H*_ch_ data (Hadži et al., 2017b). The HigA2_3–23_ folding enthalpy is then estimated by summing the enthalpy contributions of residues that adopt helix conformation in the bound-state resulting in Δ*H*_fold_ = −11.9 kcal mol^−1^. Alternatively, we also used the amino acid Δ*H*_ch_ values obtained from the temperature dependence of the helix propensities of random copolymers with different guest amino acids to estimate Δ*H*_fold_ of HigA2_3–23_ (Scheraga, 1978). Using Scheraga’s dataset (Poland, 2002) we estimated Δ*H*_fold_ = −10.5 kcal mol^−1^, in close agreement with the estimate based on Richardson's dataset. We consider the two Δ*H*_fold_ estimates as the upper and lower bounds and use the mean value −11.2 kcal mol^−1^ as the most likely value for Δ*H*_fold_.

**FIGURE 2:**
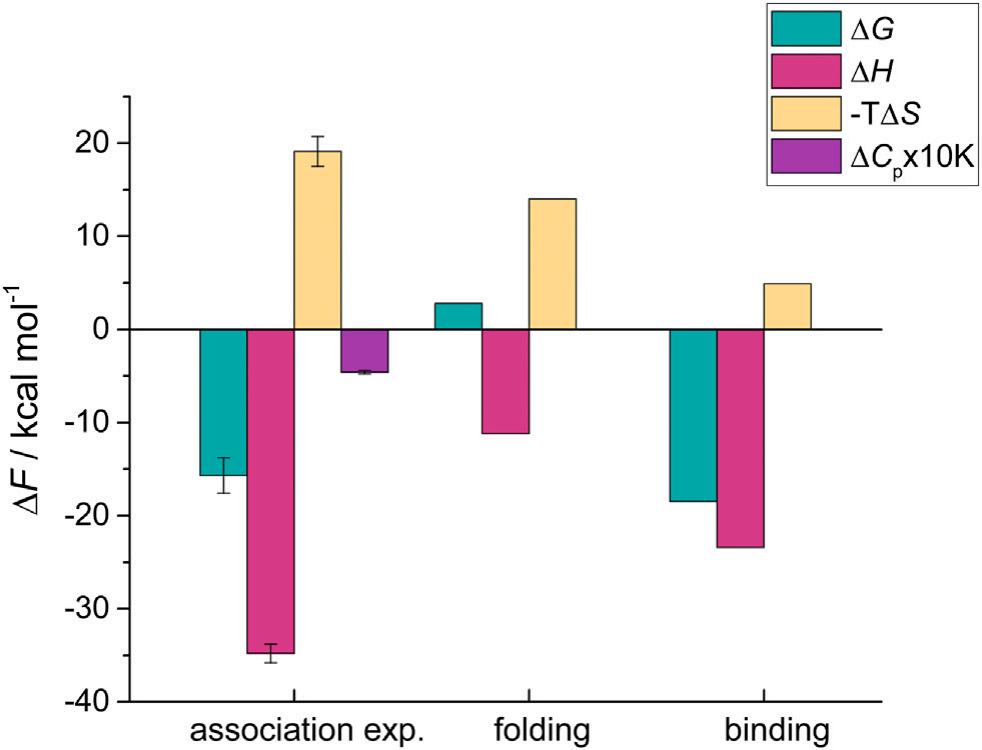
Decomposition of the thermodynamic contributions accompanying HigA2_3−23_-HigB2 association into the folding and binding contributions. The overall thermodynamic parameters for association were determined using the competition ITC experiments and are shown in first set of bars on the left (association exp.). The folding contributions (middle) were estimated from the LR model and were subtracted from the overall values to obtain the binding contributions, shown on the right. Parameters are reported at T = 25°C.

Taken together, these results show that folding of HigA2_3-23_ from the intrinsically disordered state with low helicity into the folded, helical bound-state conformation is associated with the following thermodynamic contributions: Δ*G*_fold_ = 2.8 kcal mol^−1^, Δ*H*_fold_ = −11.2 kcal mol^−1^, and a large entropic penalty −TΔ*S*_fold_ = 14.0 kcal mol^−1^ (Figure 2). By subtracting the folding contribution from the overall values of the IDP-target association we obtain the corresponding binding contributions (Δ*F*_bind_ = Δ*F*_assoc_ - Δ*F*_fold_; Δ*F* = Δ*G*, Δ*H*, Δ*S*). The binding of HigA2_3–23_ is strongly favored Δ*G*_bind_ = −18.5 kcal mol^−1^ and it significantly outweighs the unfavorable folding contribution. This is due to a strongly favorable negative enthalpy change Δ*H*_bind_ = −23.4 kcal mol^−1^, reflecting the formation of strong intermolecular contacts with its target (Figure 2, Supplementary Table S1). In contrast, HigA2_3–23_ binding is entropically unfavorable −TΔ*S*_bind_ = 4.9 kcal mol^−1^, despite the burial of a fairly large hydrophobic surface area which is generally associated with favorable desolvation entropy. This indicates that the ultra-high affinity of HigA2_3–23_-HigB2 interaction is achieved by a strong optimization of IDP-target interactions, resulting in very high magnitudes of the binding contributions (Δ*G*_bind_, Δ*H*_bind_) that can compensate for the unfavorable folding terms.

### Energetic Optimization of the HigA2-HigB2 Binding Interface Is Comparable to the One Observed for the Association of Globular Proteins

**FIGURE 3:**
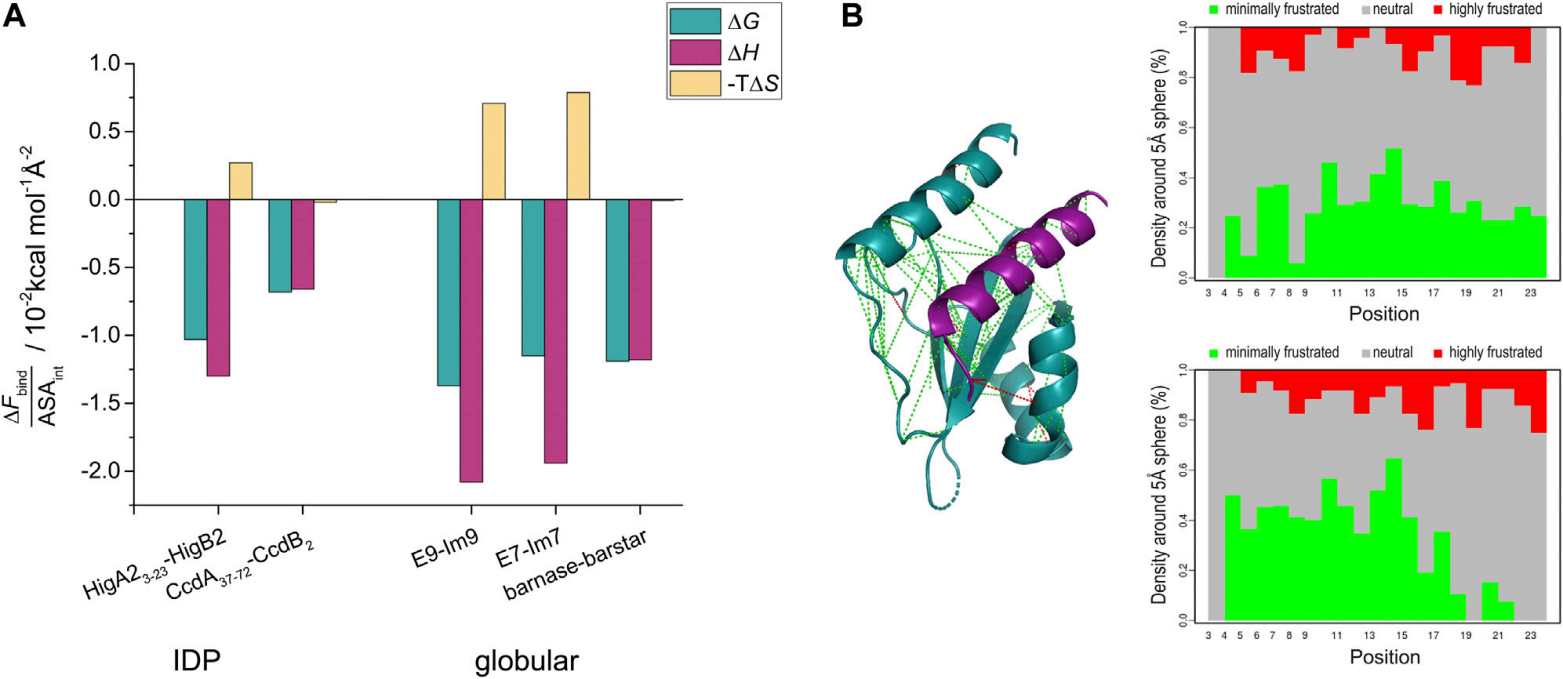
Interaction surface optimization and frustration analysis. **(A)** The interface surface-area normalized binding contributions (Δ*F*_bind_/ASA_int_) of ultra-high affinity complexes from IDPs (HigA2_3-23_-HigB2 and CcdA_37-72_-CcdB_2_) and from globular proteins. Thermodynamic parameters for binding (folding is subtracted from the overall values in case of IDPs) were normalized per interface surface area (ASA_int_) and are shown as bars and reported at T = 25°C. **(B)** Frustration index analysis of HigA2_3-23_ in complex with the target. Inter-residue interactions are shown on the structure of the HigA2_3-23_-HigB2 complex with lines colored according to frustration index (green no frustration, red frustrated interactions). The values of frustration index for HigA2_3-23_ are presented on the right panels: upper shows the conformational frustration index, while the bottom one shows the mutational frustration index (Parra et al., 2016).

Next, we examine how the binding energetics of HigA2_3-23_-HigB2 compares to other ultra-high affinity complexes formed by globular proteins. These include well-known examples such as the barnase-barstar and the complexes between various immunity proteins and colicin-DNases (Hartley, 1989; Keeble et al., 2006). The overall Gibbs free energies of globular high affinity complexes (Δ*G*_assoc_ about −19 kcal/mol) are approximately 3 kcal/mol more negative compared to HigA2_3–23_-HigB2. Since no significant conformational changes occur upon formation of globular high affinity complexes in any of these proteins (Δ*G*_fold_ = 0) these association parameters can be considered to reflect binding contributions. The observed higher affinities for globular complexes are either due to more favorable entropic contribution (as in the case of barnase-barstar) or more favorable enthalpic contributions (E7-Im7 and E9-Im9) (Supplementary Table S1). However, as shown above, the Δ*G*_assoc_ of IDP-target complex is strongly affected by the IDP folding contribution. Accordingly, a comparison of Δ*G*_bind_ contribution reveals a much smaller, 0.5 kcal/mol difference between HigA2_3–23_-HigB2 and pairs of globular proteins (Supplementary Table S1). In other words, for a hypothetical process in which the HigA2_3–23_ peptide would be prefolded (Δ*G*_fold_ is zero), the affinity would reach *K*_D_ = 30 fM, which is same order of magnitude as the affinities of the globular ultra-high affinity complexes (for example *K*_D_ for barnase-barstar is 10 fM). Since all of these complexes differ somewhat in size, we also compared the energetic contributions normalized to the interfacial surface area (ASA_int_) (Figure 3A, Supplementary Table S1). The surface normalized free energy of HigA2_3-23_-HigB2 binding, Δ*G*_bind_ /ASA_int_ = −1.03 × 10^−2^ kcal mol^−1^ Å^−2^ is only slightly less favorable than that of barnase-barstar interaction (−1.11 × 10^−2^ kcal mol^−1^ Å^−2^). This is due to the difference in entropic contribution (unfavorable for HigA2-HigB2, neutral for barnase-barstar) - Figure 3A. Similarly, the normalized binding free energy is less favorable compared to E9-Im9 and E7-Im7 (Figure 3A).

Of particular interest is the comparison of enthalpic binding contributions, which directly reflect the strength of IDP-target interactions. The average surface-normalized binding enthalpy for the ultra-high affinity globular binders is Δ*H*_bind_ /ASA_int_ = −1.7 × 10^−2^ kcal mol^−1^ Å^−2^, which does not significantly differ from the value for HigA2_3–23_-HigB2 (−1.3 × 10^−2^ kcal mol^−1^ Å^−2^). To put these values in perspective, we compared them with the ones calculated for the annotated database of heteromeric dimers by Kastritis et al. (Kastritis et al., 2011). For 27 protein-protein complexes characterized by ITC the normalized enthalpy contribution ranges from −0.05 × 10^−2^ to −1.95 × 10^−2^ kcal mol^−1^ Å^−2^ with the average value of −0.62 × 10^−2^ kcal mol^−1^ Å^−2^. This indicates that the ultra-high affinity binders discussed above achieve very high values of Δ*H*_bind_ /ASA_int_ and suggests that this metric can be used to assess the degree of energy optimization of the binding interface. Thus, the surface-normalized binding enthalpy for the HigA2_3–23_-HigB2 binding is more than twice the average of the Kastritis data set of heterodimeric proteins and even higher than that for the barnase-barstar system (Δ*H*_bind_ /ASA_int_ = −1.1 × 10^−2^ kcal mol^−1^ Å^−2^). This suggests that the HigA2_3–23_-HigB2 binding interface is highly energy optimized, to an extent which is comparable to the interfaces of globular ultra-high affinity binders.

## Discussion

In this work, we present a detailed thermodynamic analysis of an association with ultra-high affinity (*K*_D_ = 3 pM) mediated by an IDP undergoing a transition from disorder to order. Such high affinities are rarely observed in protein associations, especially in the interactions involving IDPs. In general, IDP binding affinities are considered to be moderate, often in the micromolar range (Huang and Liu, 2013; Teilum et al., 2015). It has been argued that IDP folding lowers association affinity while the binding specificity remains unaffected, resulting in uncoupling of binding affinity and specificity, which enables polyspecific IDP interactions (Dunker et al., 2001). A recent understanding suggests that IDPs can often achieve moderate to high affinities (nanomolar range) and retain high specificity (Gianni and Jemth, 2019). A review of association affinities has shown that the average Gibbs free energy value of association between a globular and an IDP protein are −8.5 kcal mol^−1^, while the value for two globular proteins is −11.1 kcal mol^−1^ (Teilum et al., 2015). The study suggested that the difference of 2.5 kcal mol^−1^ between the two groups is due to the entropic penalty of IDP folding.

**FIGURE 4:**
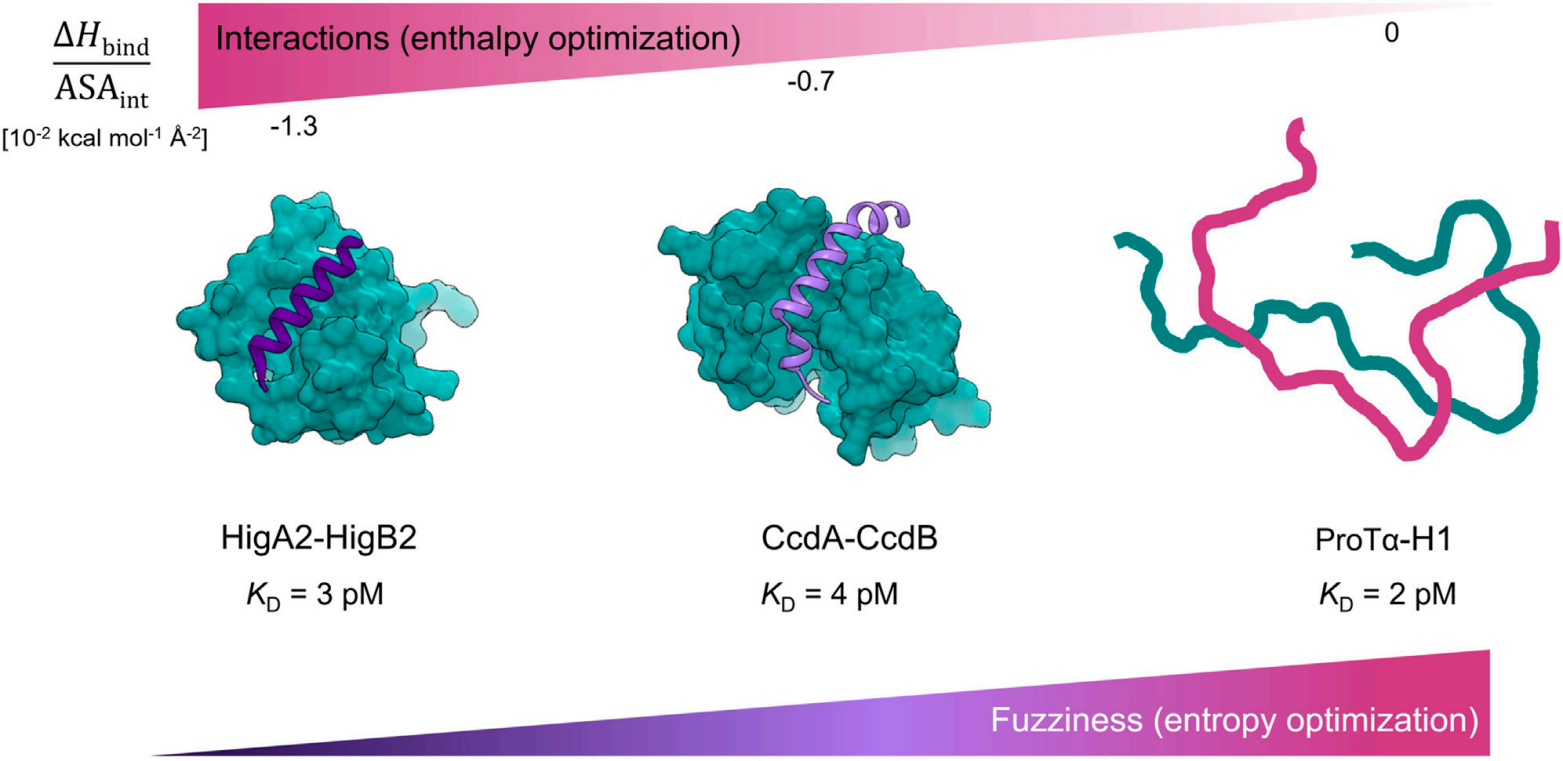
Ultra-high affinity association of IDPs reaching picomolar affinities employ different thermodynamic strategies. Complexes are ordered according to the degree of energy optimization as estimated by the Δ*H*_bind_/ASA_int_ values. For the HigA2_3-23_-HigB2 complex the degree of energy optimization is similar to one observed for the pairs of globular high affinity binders (Supplementary Table S1, Figure 3A). The CcdA_37-72_-CcdB_2_ complex exhibits fuzziness in the bound-state (Hadži et al., 2017b) and has lower degree of energy optimization (Supplementary Table S1, Figure 3A). The high affinity complex between ProTα and histone H1 is highly dynamic and is entropically stabilized (Borgia et al., 2018).

Nonetheless, a handful of examples of IDP binders with ultra-high affinity have been characterized to date. To the best of our knowledge, these are: intrinsically disordered CcdA that binds to the gyrase poison CcdB (Δ*G*_assoc_ = −15.6 kcal mol^−1^, *K*_D_ = 4 pM) (Drobnak et al., 2013), HigA2 binding to the mRNAse HigB2 (Δ*G*_assoc_ = −15.7 kcal mol^−1^, *K*_D_ = 3 pM), presented here, and the interaction between the intrinsically disordered histone H1 and the nuclear chaperone prothymosin α (Δ*G*_assoc_ = −16.0 kcal mol^−1^, *K*_D_ = 2 pM) (Borgia et al., 2018) – Figure 4. Interestingly, these three complexes appear to use completely different mechanisms to achieve ultra-tight binding, as reflected by different degrees of disorder in the bound state. For HigA2, the strategy involves strong optimization of the IDP-target contacts, high surface complementarity and formation of a relatively ordered complex. As such, this strategy shares many similarities with that observed for the globular high affinity binders, such as in barnase-barstar complex. For example, both types of complexes have a small interaction surface area (1800 Å^2^ for HigA2_3–23_-HigB2 and 1700 Å^2^ for barnase-barstar), comparable binding enthalpy (−23 kcal mol^−1^ and −19 kcal mol^−1^), and a similar surface-normalized free energy and enthalpy contributions (Figure 3A, Supplementary Table S1). This indicates that the binding interactions are highly optimized, which is also consistent with the analysis of the frustration indices (Figure 3B). The interacting residues of HigA2-HigB2 are neutral with respect to frustration level, as indicated by a positive mean frustration index, and have overall similar frustration levels as the interactions in the globular high affinity binders (Supplementary Figure S5 and Supplementary Figure S6). The average frustration indexes for the interface residues are 0.33/0.33 (mutational/conformational) for the globular complexes (E7-Im7 and barnase-barstar) and 0.53/0.45 (mutational/conformational) for the HigA2-HigB2 IDP-target complex. This shows that although IDP-target complexes generally have more frustrated IDP-target interactions (Freiberger et al., 2021), exceptions to this trend exist and allow the formation of ultra-high affinity complexes.

The interaction between intrinsically disordered CcdA (high affinity helix folding/binding fragment: CcdA_37-72_) and its globular target CcdB_2_ is also accompanied by CcdA folding into α-helix, but the helix is partially unstructured and kinked (Figure 4). The CcdA_37–72_-CcdB_2_ interface is much larger (2850 Å^2^) compared to that of HigA2_3–23_-HigB2. The overall thermodynamic parameters (association) are similar to those accompanying HigA2_3–23_-HigB2 association, but due to the larger surface area, the per area normalized contributions are smaller (Supplementary Table S1). We have previously shown that while CcdA_37–72_-CcdB_2_ forms a high affinity complex, the target-bound CcdA_37–72_ retains a considerable dynamics and exhibits fuzziness that largely determines the properties of CcdA-CcdB association (Hadži et al., 2017b). Compared to the HigA2_3–23_-HigB2, the complex appears to be more dynamic and less optimized in terms of enthalpy but more optimized in terms of entropy, most likely due to the bound-state fuzziness (Figure 3A). The final example of an ultra-high affinity interaction is the association between histone H1 and prothymosin α (ProTα), which reaches 2 pM affinity. The complex is highly dynamic, involving a larger interface compared to the systems discussed above. The interaction appears to be mediated entirely by electrostatics and does not involve heat exchange, hence the Δ*H*_bind_/ASA_int_ can be assumed to be zero (Figure 4). Overall, all three systems can achieve ultra-high, picomolar affinity via different strategies. In the case of the most ordered IDP system HigA2-HigB2 described here, we show that the magnitudes of binding contributions to the energetics of IDP-target association are comparable to those for binding of the high affinity globular proteins.

In summary, the dissection of thermodynamics of protein association coupled with protein folding allowed us to explore the thermodynamic origin of ultra-high affinity association of IDPs and compare it to high affinity binding of globular proteins. In particular, the enthalpic term is strongly affected by the IDP folding, precluding a direct use of the overall association parameters, thus underpinning the need of decomposed values to make a meaningful comparison between interactions mediated by globular proteins and IDPs. We have shown that the binding thermodynamics of HigA2-HigB2 is comparable to that of the strongest observed binding in globular proteins, despite the unfavorable IDP folding. While the HigA2-HigB2 interaction appears to resemble that of ultra-high affinity globular protein pairs, particularly with respect to enthalpy optimization of the IDP-target interface, other strategies that rely on entropy optimization through fuzziness could just as well lead to the ultra-tight binding. It would be of considerable interest to understand whether there are some additional biological implications associated with a particular thermodynamic strategy for achieving ultra-tight binding. For example, recent data for the association between ProTα and H1 indicate that this entropy-driven fuzzy, electrostatic interaction is marked by rapid kinetics (Sottini et al., 2020). This enables a fast exchange of the associated partners in the complex and facilitates formation of ternary complexes. On the other hand, toxin-antitoxin and colicin-inhibitor complexes are highly specific, which is likely related to the enthalpic optimization of interaction in these complexes. Thus, one may speculate that the selection of a thermodynamic strategy for achieving ultra-tight binding is further linked to such kinetic and specificity requirements, that are best suited for fulfilling a biological task of the complex.

## Materials and Methods

### Materials

Proteins (HigA2, HigB2 and nanobodies) were prepared as described in previous work (Hadži et al., 2017a). Peptides HigA2_3–23_ and HigA2_3–33_ were obtained from China Peptides Co., Ltd. and were at least 95% pure. Both peptides contain an additional tryptophan residue at their C-terminus that enables concentration determination by UV spectrophotometry. Concentrations of peptides and proteins were determined by measuring absorbance at 280 nm and using following molar extinction coefficients (in M^−1^ cm^−1^): 15930 (HigB2), 5500 (full-length HigA2, monomer), 5690 (HigA2_3–23_ and HigA2_3–33_).

### Determination of the HigA2_3-23_ Folding Free Energy

The helix propensity of HigA2_3–23_ peptide was estimated using the Lifson-Roig model of helix-coil transition (Lifson and Roig, 1961). The model enumerates all possible configurations of the polypeptide chain and assumes that every single residue can occupy either helix (h) or coil (c) state. The analytical expression for the partition function Q is obtained using the matrix method. Two parameters define the partition function: nucleation parameter *v* and propagation parameter *w*. Nucleation parameter *v* is assumed to be constant (*v* = 0.048), mainly entropic by origin and therefore temperature-independent, while the helix propagation parameter *w* is related to the formation of helix hydrogen bonds and is considered as temperature-dependent. From the partition function Q many useful properties of the system can be calculated (Poland and Scheraga, 1970). One such property is the fractional helicity (f_H_), which is related to the average number of *w*-weighted residues (<n_h_>) and a maximal-length helix, which is N-2 for a polypeptide chain with N residues. Using Eq. 1 for f_H_, we can compare the experimental data with the LR model prediction, since fractional helicity can be evaluated experimentally by measuring ellipticity using CD spectroscopy (Eq. 3).

$${\text{f}}_{\text{H}}=\frac{<{\text{n}}_{\text{h}}>}{\text{N}-2}=\frac{1}{\left(\text{N}-2\right)}\frac{\partial \text{ lnQ}}{\partial \ln w}$$ (1)

To estimate folding free energy of HigA2_3-23_ we first use the above equation to obtain the value of helix propagation parameter *w* which gives the best agreement between experimental (Eq. 3) and model calculated (Eq. 1) fractional helicity. We then evaluate the partition function of the unbound peptide (Q_unbound_). The partition function of the bound-state peptide (Q_bound_) is evaluated by assuming that all the residues in helical conformation (as evaluated from the crystal structure) are weighted by the experimentally derived *w* parameter. In other words, only residues at the helix termini are not constrained to the helical state and therefore contribute to the conformational heterogeneity of the bound-state HigA2_3–23_ ensemble. Again, a numerical evaluation is performed using parameter *w* as determined earlier on the unbound ensemble, leading to Q_bound_. The free energy associated with a transition (folding) of the unbound HigA2_3–23_ to its folded bound-state conformation is calculated from the ratio of partition functions Q_unbound_ and Q_bound_ as:

$$\text{Δ}G_{\mathrm{fold}}=-\text{RT}\ln\frac{{\text{Q}}_{\text{bound}}}{{\text{Q}}_{\text{unbound}}}$$ (2)

### Isothermal Titration Calorimetry

Prior to titration all the samples were extensively dialyzed against 20 mM sodium phosphate pH 7.5, 150 mM NaCl buffer. All titrations were performed on the VP-ITC (Microcal, United States) at 25°C expect the ones used for Δ*C*_p_ determination (Supplementary Figure S7). Competition ITC experiment consists of two subsequent ITC titrations. In the first titration “weak” binder (nanobody) is titrated to the toxin to high molar ratio (excess) to fully saturate toxin's binding site. Next, the second titration involves a competition where antitoxin or its truncated version is titrated into the product of the first titration (nanobody-HigB2 complex). During this titration high affinity antitoxin competes with nanobody for the binding site on the toxin and displaces nanobody from the toxin. Concentration of HigB2 toxin was 4 µM in all of the ITC titrations, whereas concentrations of the ligands (nanobodies, HigA2 protein/peptides) were chosen to achieve appropriate final molar ratio of titration: 104.7 µM for nanobodies and 46.5 µM for HigA2 protein/peptides. Integration of raw ITC thermogram was done using NITPIC (Scheuermann and Brautigam, 2015). Fitting procedure was accomplished using Sedphat and in-built models for 1:1 binding and competitive binding experiments. Errors reported were determined by Sedphat’s built-in error propagation analysis toolkit to estimate a confidence interval (automated method to explore the error surface of the fit using F-statistics) (Zhao et al., 2015; Brautigam et al., 2016). Experiments were performed in three replicates using different batches of proteins.

### CD Spectroscopy

Circular dichroism measurements were carried out on Jasco J-1500 CD spectrophotometer at 25°C in a cuvette with 1 mm optical path length. All data were measured in 20 mM sodium phosphate pH 7.5, 150 mM NaCl buffer. Concentrations of macromolecules in CD experiments were 40 µM for HigA2_3–23_ (Supplementary Figure S4) and 20 µM for HigA2_3–23_-HigB2 complex (Supplementary Figure S3B). Signal at 222 nm in mean residue ellipticity was used to estimate fractional helicity of the unbound peptide, using conventionally used temperature baselines for fully helical and coil state (Luo and Baldwin, 1997).

$${\text{f}}_{\mathrm{H,}\exp}=\frac{[\text{θ}]-[\text{θ}{]}_{c}}{[\text{θ}{]}_{h}-[\text{θ}{]}_{c}}$$ (3)

### Structure-based Calculations

Accessible surface areas (ASAs) were calculated with NACCESS using a water radius of 1.4 Å (Hubbard and Thornton, 1993). Interaction surface area (ASA_int_) was calculated from the structure of the complex as difference between ASA of the complex and sum of ASAs for both components in the complex. Structures of complexes used for calculation are listed in Supplementary Table S1. Frustration index calculations were performed with Frustratometer server (http://frustratometer.qb.fcen.uba.ar/), using default settings and enabled electrostatics (k = 4.15) (Parra et al., 2016). A residue with an average frustration index higher than 0.78 is considered as minimally frustrated, since potential structures with mutated amino acid pairs would in majority result in a less stable complexes, therefore the native pair has favorable contribution to the affinity (green line on Supplementary Figure S5 and Supplementary Figure S6). On the other end, a residue with an average frustration index lower than −1 is considered to be highly frustrated, since majority of mutations would lead to a more stable structure (red line on Supplementary Figure S5 and Supplementary Figure S6). Residues in between are marked as neutral (Ferreiro et al., 2007). Only the interacting residues were considered in the final analysis shown on Supplementary Figure S5 and Supplementary Figure S6. Structures used for calculation are listed in Supplementary Table S1.

## Data Availability

The original contributions presented in the study are included in the article/Supplementary Material, further inquiries can be directed to the corresponding authors.

## References

[B1] Borgia A. Borgia M. B. Bugge K. Kissling V. M. Heidarsson P. O. Fernandes C. B. (2018). Extreme Disorder in an Ultrahigh-Affinity Protein Complex. Nature 555, 61–66. 10.1038/nature25762 29466338 PMC6264893

[B2] Brautigam C. A. Zhao H. Vargas C. Keller S. Schuck P. (2016). Integration and Global Analysis of Isothermal Titration Calorimetry Data for Studying Macromolecular Interactions. Nat. Protoc. 11, 882–894. 10.1038/nprot.2016.044 27055097 PMC7466939

[B3] Chothia C. Janin J. (1975). Principles of Protein-Protein Recognition. Nature 256, 705–708. 10.1038/256705a0 1153006

[B4] Conte L. L. Chothia C. Janin J. (1999). The Atomic Structure of Protein-Protein Recognition Sites 1. J. Mol. Biol. 285, 2177–2198. 10.1006/jmbi.1998.2439 9925793

[B5] Drobnak I. De Jonge N. Haesaerts S. Vesnaver G. Loris R. Lah J. (2013). Energetic Basis of Uncoupling Folding from Binding for an Intrinsically Disordered Protein. J. Am. Chem. Soc. 135, 1288–1294. 10.1021/ja305081b 23289531

[B6] Dunker A. K. Lawson J. D. Brown C. J. Williams R. M. Romero P. Oh J. S. (2001). Intrinsically Disordered Protein. J. Mol. Graphics Model. 19, 26–59. 10.1016/S1093-3263(00)00138-8 11381529

[B7] Ferreiro D. U. Hegler J. A. Komives E. A. Wolynes P. G. (2007). Localizing Frustration in Native Proteins and Protein Assemblies. Proc. Natl. Acad. Sci. 104, 19819–19824. 10.1073/PNAS.0709915104 18077414 PMC2148382

[B8] Flock T. Weatheritt R. J. Latysheva N. S. Babu M. M. (2014). Controlling Entropy to Tune the Functions of Intrinsically Disordered Regions. Curr. Opin. Struct. Biol. 26, 62–72. 10.1016/j.sbi.2014.05.007 24930020

[B9] Freiberger M. I. Wolynes P. G. Ferreiro D. U. Fuxreiter M. (2021). Frustration in Fuzzy Protein Complexes Leads to Interaction Versatility. J. Phys. Chem. B 125, 2513–2520. 10.1021/acs.jpcb.0c11068 33667107 PMC8041309

[B10] Fuxreiter M. (2019). Fold or Not to Fold upon Binding - Does it Really Matter? Curr. Opin. Struct. Biol. 54, 19–25. 10.1016/j.sbi.2018.09.008 30340123

[B11] Gianni S. Freiberger M. I. Jemth P. Ferreiro D. U. Wolynes P. G. Fuxreiter M. (2021). Fuzziness and Frustration in the Energy Landscape of Protein Folding, Function, and Assembly. Acc. Chem. Res. 54, 1251–1259. 10.1021/acs.accounts.0c00813 33550810 PMC8023570

[B12] Gianni S. Jemth P. (2019). Affinity Versus Specificity in Coupled Binding and Folding Reactions. Protein Eng. Des. Sel. 32, 355–357. 10.1093/protein/gzz020 31397874

[B13] Hadži S. Garcia-Pino A. Haesaerts S. Jurėnas D. Gerdes K. Lah J. (2017a). Ribosome-Dependent Vibrio cholerae mRNAse HigB2 Is Regulated by a β-strand Sliding Mechanism. Nucleic Acids Res. 45, 4972–4983. 10.1093/nar/gkx138 28334932 PMC5416850

[B14] Hadži S. Mernik A. Podlipnik Č. Loris R. Lah J. (2017b). The Thermodynamic Basis of the Fuzzy Interaction of an Intrinsically Disordered Protein. Angew. Chem. Int. Ed. 56, 14494–14497. 10.1002/anie.201707853 28914483

[B15] Hartley R. W. (1989). Barnase and Barstar: Two Small Proteins to Fold and Fit Together. Trends Biochem. Sci. 14, 450–454. 10.1016/0968-0004(89)90104-7 2696173

[B16] Huang Y. Liu Z. (2013). Do intrinsically Disordered Proteins Possess High Specificity in Protein-Protein Interactions? Chem. Eur. J. 19, 4462–4467. 10.1002/chem.201203100 23436397

[B17] Hubbard S. J. Thornton J. M. (1993). “Naccess”, Computer Program. London: Department of Biochemistry and Molecular Biology, University College London.

[B18] Jones S. Thornton J. M. (1996). Principles of Protein-Protein Interactions. Proc. Natl. Acad. Sci. 93, 13–20. 10.1073/pnas.93.1.13 8552589 PMC40170

[B19] Kastritis P. L. Moal I. H. Hwang H. Weng Z. Bates P. A. Bonvin A. M. J. J. (2011). A Structure-Based Benchmark for Protein-Protein Binding Affinity. Protein Sci. 20, 482–491. 10.1002/pro.580 21213247 PMC3064828

[B20] Keeble A. H. Kirkpatrick N. Shimizu S. Kleanthous C. (2006). Calorimetric Dissection of Colicin DNase−Immunity Protein Complex Specificity. Biochemistry 45, 3243–3254. 10.1021/bi052373o 16519519

[B21] Kleanthous C. Hemmings A. M. Moore G. R. James R. (1998). Immunity Proteins and Their Specificity for Endonuclease Colicins: Telling Right from Wrong in Protein-Protein Recognition. Mol. Microbiol. 28, 227–233. 10.1046/j.1365-2958.1998.00811.x 9622349

[B22] Ladbury J. E. Klebe G. Freire E. (2010). Adding Calorimetric Data to Decision Making in lead Discovery: A Hot Tip. Nat. Rev. Drug Discov. 9, 23–27. 10.1038/nrd3054 19960014

[B23] Li G.-Y. Zhang Y. Inouye M. Ikura M. (2009). Inhibitory Mechanism of *Escherichia coli* RelE-RelB Toxin-Antitoxin Module Involves a Helix Displacement Near an mRNA Interferase Active Site. J. Biol. Chem. 284, 14628–14636. 10.1074/JBC.M809656200 19297318 PMC2682910

[B24] Lifson S. Roig A. (1961). On the Theory of Helix-Coil Transition in Polypeptides. J. Chem. Phys. 34, 1963–1974. 10.1063/1.1731802

[B25] Luo P. Baldwin R. L. (1997). Mechanism of Helix Induction by Trifluoroethanol: A Framework for Extrapolating the Helix-Forming Properties of Peptides from Trifluoroethanol/water Mixtures Back to Water. Biochemistry 36, 8413–8421. 10.1021/bi9707133 9204889

[B26] Nick Pace C. Martin Scholtz J. (1998). A helix Propensity Scale Based on Experimental Studies of Peptides and Proteins. Biophysical J. 75, 422–427. 10.1016/s0006-3495(98)77529-0 PMC1299714 9649402

[B27] Pancsa R. Fuxreiter M. (2012). Interactions via Intrinsically Disordered Regions: What Kind of Motifs? IUBMB Life 64, 513–520. 10.1002/iub.1034 22535488

[B28] Parra R. G. Schafer N. P. Radusky L. G. Tsai M.-Y. Guzovsky A. B. Wolynes P. G. (2016). Protein Frustratometer 2: A Tool to Localize Energetic Frustration in Protein Molecules, Now with Electrostatics. Nucleic Acids Res. 44, W356–W360. 10.1093/nar/gkw304 27131359 PMC4987889

[B29] Poland D. (2002). Contribution of Secondary Structure to the Heat Capacity and Enthalpy Distribution of the Unfolded State in Proteins. Biopolymers 63, 59–65. 10.1002/bip.1062 11754348

[B30] Poland D. Scheraga H. A. (1970). Theory of Helix-Coil Transitions in Biopolymers; Statistical Mechanical Theory of Order-Disorder Transitions in Biological Macromolecules. New York: Academic Press.

[B31] Richardson J. M. Lopez M. M. Makhatadze G. I. (2005). Enthalpy of Helix-Coil Transition: Missing Link in Rationalizing the Thermodynamics of Helix-Forming Propensities of the Amino Acid Residues. Proc. Natl. Acad. Sci. 102, 1413–1418. 10.1073/pnas.0408004102 15671166 PMC547846

[B32] Scheraga H. A. (1978). Use of Random Copolymers to Determine the Helix-Coil Stability Constants of the Naturally Occurring Amino Acids. Pure Appl. Chem. 50, 315–324. 10.1351/pac197850040315

[B33] Scheuermann T. H. Brautigam C. A. (2015). High-Precision, Automated Integration of Multiple Isothermal Titration Calorimetric Thermograms: New Features of NITPIC. Methods 76, 87–98. 10.1016/j.ymeth.2014.11.024 25524420 PMC4380771

[B34] Sottini A. Borgia A. Borgia M. B. Bugge K. Nettels D. Chowdhury A. (2020). Polyelectrolyte Interactions Enable Rapid Association and Dissociation in High-Affinity Disordered Protein Complexes. Nat. Commun. 11, 1–14. 10.1038/s41467-020-18859-x 33184256 PMC7661507

[B35] Sugase K. Dyson H. J. Wright P. E. (2007). Mechanism of Coupled Folding and Binding of an Intrinsically Disordered Protein. Nature 447, 1021–1025. 10.1038/nature05858 17522630

[B36] Sukhwal A. Sowdhamini R. (2013). Oligomerisation Status and Evolutionary Conservation of Interfaces of Protein Structural Domain Superfamilies. Mol. Biosyst. 9, 1652–1661. 10.1039/c3mb25484d 23532342

[B37] Teilum K. Olsen J. G. Kragelund B. B. (2015). Globular and Disordered-The Non-Identical Twins in Protein-Protein Interactions. Front. Mol. Biosci. 2, 40. 10.3389/fmolb.2015.00040 26217672 PMC4496568

[B38] Tompa P. Fuxreiter M. (2008). Fuzzy Complexes: Polymorphism and Structural Disorder in Protein-Protein Interactions. Trends Biochem. Sci. 33, 2–8. 10.1016/j.tibs.2007.10.003 18054235

[B39] Wood C. W. Ibarra A. A. Bartlett G. J. Wilson A. J. Woolfson D. N. Sessions R. B. (2020). BAlaS: Fast, Interactive and Accessible Computational Alanine-Scanning Using BudeAlaScan. Bioinformatics 36, 2917–2919. 10.1093/BIOINFORMATICS/BTAA026 31930404

[B40] Zhao H. Piszczek G. Schuck P. (2015). SEDPHAT - A Platform for Global ITC Analysis and Global Multi-Method Analysis of Molecular Interactions. Methods 76, 137–148. 10.1016/j.ymeth.2014.11.012 25477226 PMC4380758

