# Supplementary material for: Unraveling the Thermodynamics of Ultra-tight Binding of Intrinsically Disordered Proteins
Source: Front Mol Biosci. 2021 Aug 31;8:726824. doi: 10.3389/fmolb.2021.726824 (PMC8438204; doi:10.3389/fmolb.2021.726824)
Supplement: Supplementary file 1 [file DataSheet1.docx]

Supplementary Material

**Table S1**. Thermodynamic parameters and structural properties of the complexes. All parameters are reported at T = 25°C.

|  |  |  | Thermodynamic parameters | | | | Interaction surface | | |
| --- | --- | --- | --- | --- | --- | --- | --- | --- | --- |
|  |  |  | Δ*G* / kcal mol^-1^ | Δ*H* / kcal mol^-1^ | -TΔ*S /* kcal mol^-1^ | Δ*C*_p_ / kcal mol^-1^ K^-1^ | PDB | Total ΔASA_int_ | % apolar |
|  | HigA2_3-23_-HigB2 | association | -15.7 ± 1.9 | -34.6 ± 1.0 | 19.1 ± 2.9 | -0.46 ± 0.02 | 5jaa | 1800 | 76 |
|  |  | folding | 2.8 | -11.2 | 14.0 |  |  |  |  |
|  |  | binding | -18.5 | -23.4 | 4.9 |  |  |  |  |
|  |  | binding_surf_normalized [10^-2^ kcal mol^-1^ Å^-2^] | -1.03 | -1.30 | 0.27 |  |  |  |  |
|  | CcdA_37-72_-CcdB_2_^a^ | association | -15.6 ± 0.1 | -35.5 ± 0.2 | 19.9 ± 0.3 | -0.63 ± 0.02 | 3hpw | 2851 | 66 |
|  |  | folding | 3.8 | -16.6 | 20.4 |  |  |  |  |
|  |  | binding | -19.4 | -18.9 | -0.5 |  |  |  |  |
|  |  | binding_surf_normalized [10^-2^ kcal mol^-1^ Å^-2^] | -0.68 | -0.66 | -0.02 |  |  |  |  |
|  | E9-Im9^b^ |  | -18.6 ± 0.1 | -10.5 ± 0.4 | -8.1 ± 0.5 | -0.45 | 1mve | 1442 | 48 |
|  |  | binding_surf_normalized [10^-2^ kcal mol^-1^ Å^-2^] | -1.37 | -2.08 | 0.71 |  |  |  |  |
|  | E7-Im7^b^ |  | -19.8 | -30.3 ± 0.8 | 10.5 | -0.69 | 1mz8 | 1600 | 44 |
|  |  | binding_surf_normalized [10^-2^ kcal mol^-1^ Å^-2^] | -1.24 | -1.88 | 0.64 |  |  |  |  |
|  | barnase-barstar^c^ |  | -19 | -18.9 | -0.1 | -0.36 ± 0.02 | 2za4 | 1707 | 51 |
|  |  | binding_surf_normalized [10^-2^ kcal mol^-1^ Å^-2^] | -1.11 | -1.11 | -0.01 |  |  |  |  |

^a^(Drobnak et al., 2013)
^b^(Keeble et al., 2006)
^c^(Frisch et al., 1997)


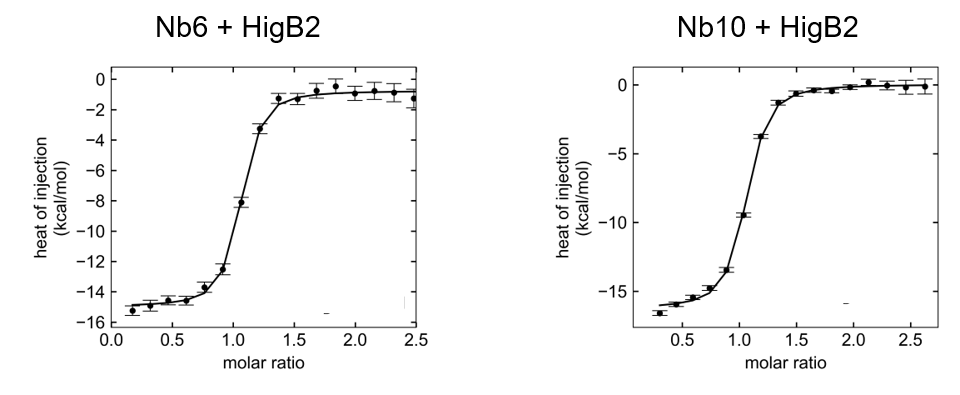


**Figure S1.** ITC binding isotherms for Nb6 (left) and Nb10 (right) to HigB2 at 25°C. Full lines represent 1:1 binding model function. Best-fit parameters are reported in Table 1 in the main text.


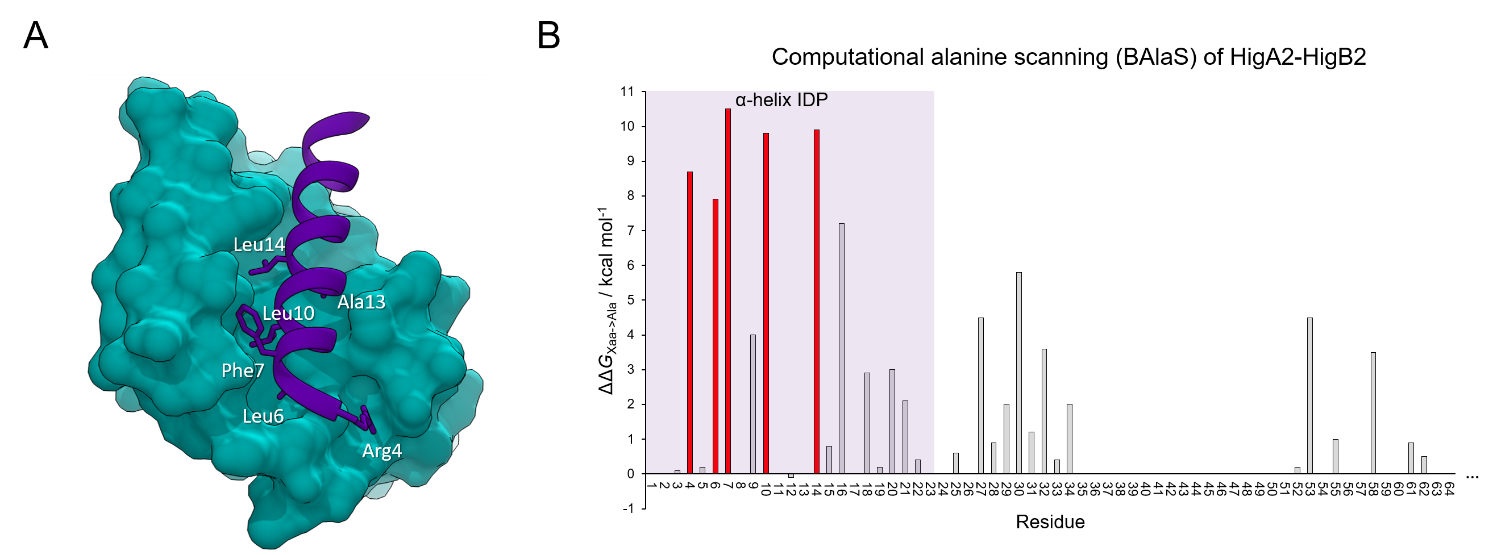


**Figure S2.** Computational analysis of HigA2-HigB2 interaction on the level of residues. **A)** Computational analysis of hotspots. Hotspot residues as identified by PPcheck on HigA2_3-23_ segment are shown as sticks (in violet). **B)** Computational alanine scanning mutagenesis. Energetic evaluation of mutations to Ala (ΔΔ*G*_Xaa->Ala_) by BAlaS was performed for residues along HigA2 sequence (Wood et al., 2020). Residues from helix forming segment of IDP, especially hotspot residues, are major contributors to the stability of the complex. Hotspot residues are shown as red bars (Ala13 not included since ΔΔ*G*(Ala13->Ala) = 0).


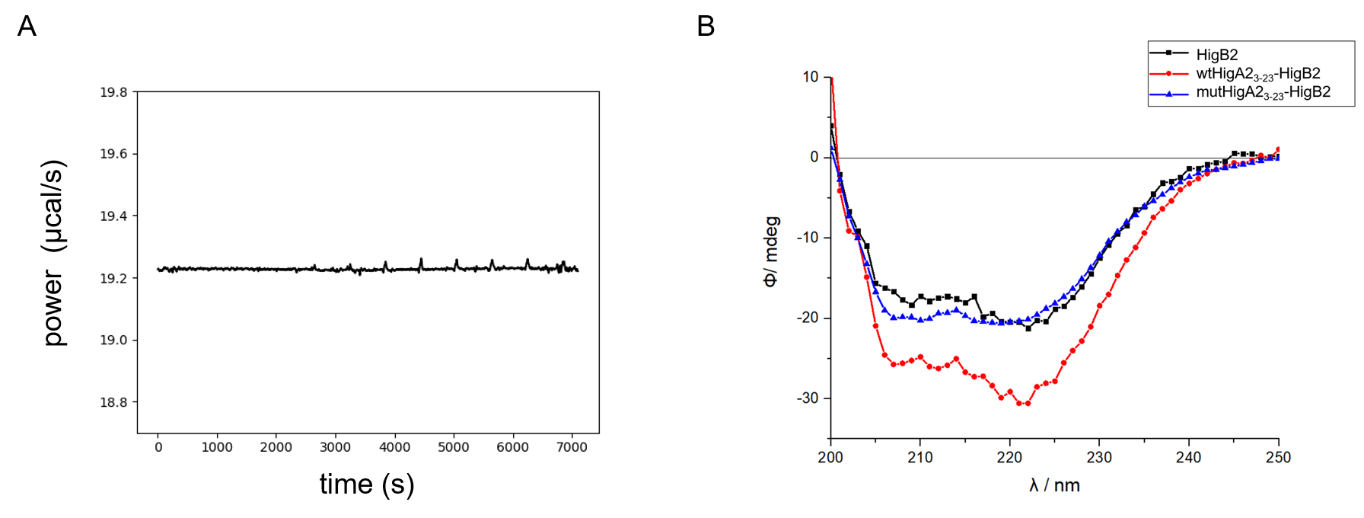

**Figure S3.** Influence of hotspot mutations on binding. No binding is observed between mutant HigA2 peptide (mutHigA2_3-23_ harboring Arg4Ala, Leu6Ala, and Phe7Ala mutations) and HigB2 toxin **A)** calorimetrically by ITC (no heat exchange) and **B)** spectroscopically by CD (no change in the α-helix content) upon addition of mutant peptide.


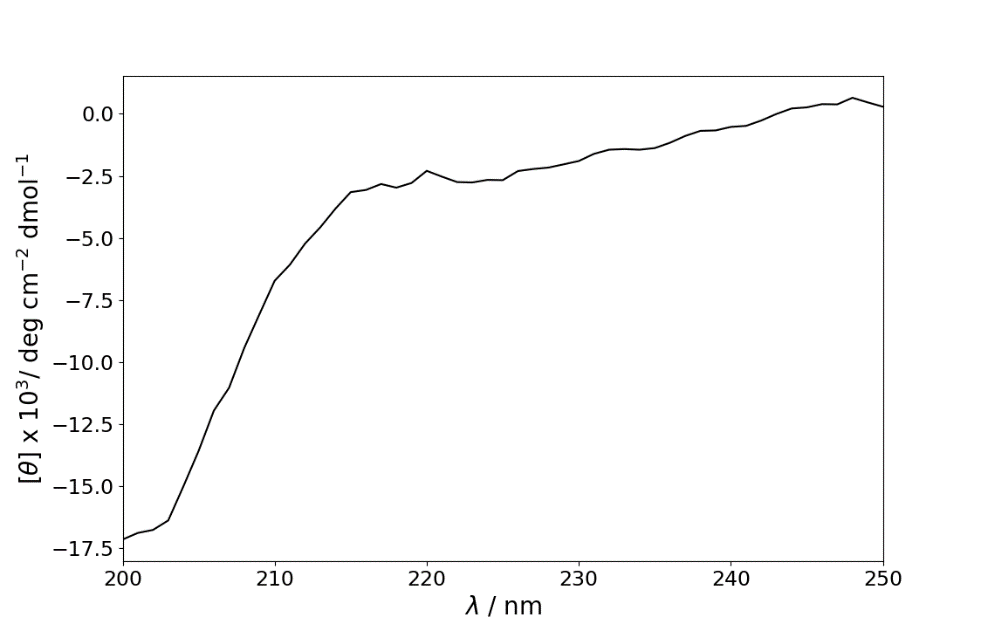


**Figure S4.** CD spectra of HigA2_3-23_ (unbound conformation) at 25°C in 20 mM phosphate buffer (pH 7.5). Some residual structure (cca. 10% helicity) is present already in unbound conformation of peptide based on the intensity of the CD signal at 222 nm and use of Equation 3 (Materials and Methods, main text).


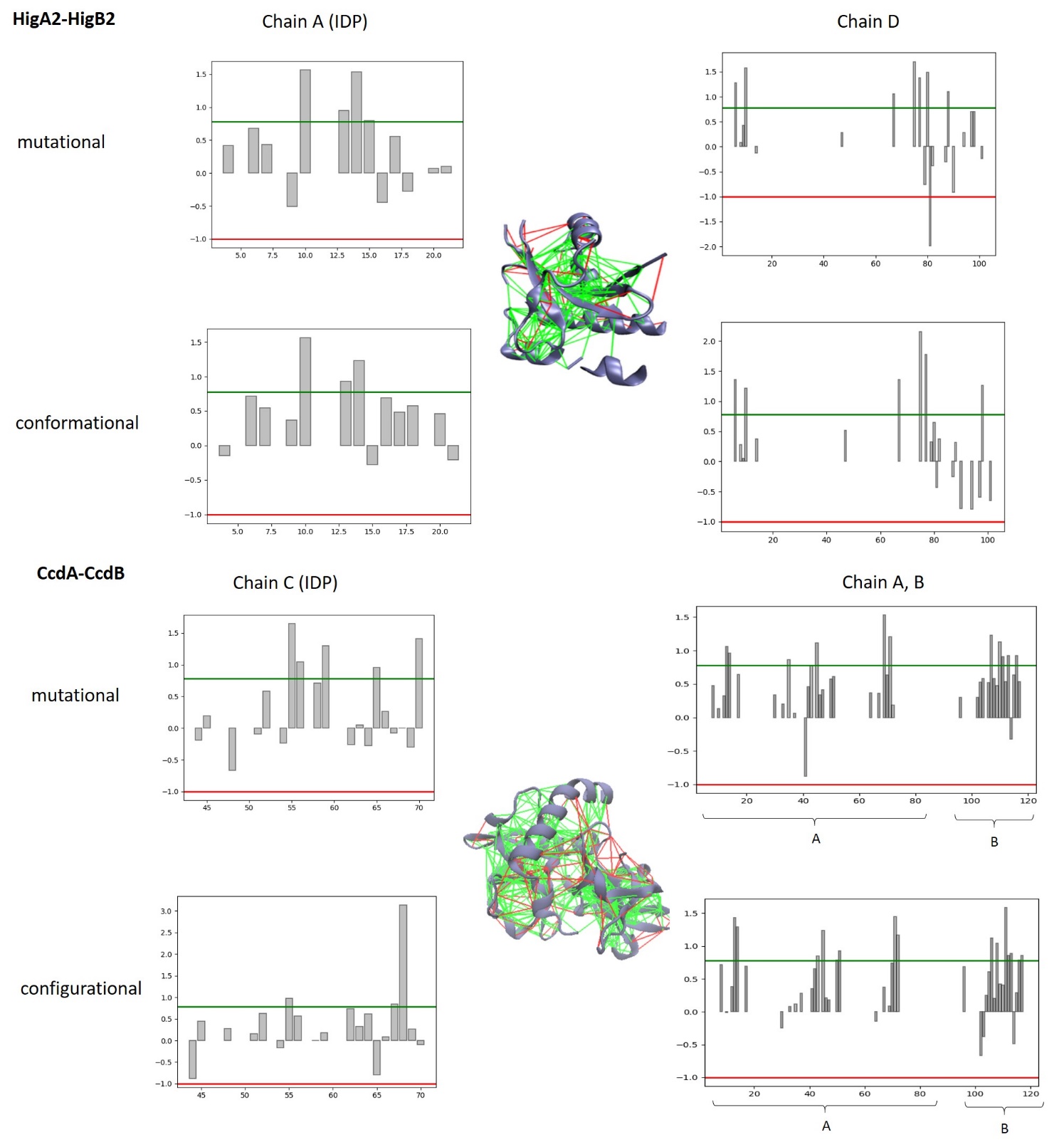


**Figure S5.** Frustration analysis of the IDP-target ultra-high affinity complexes. Frustration indices (mutational and configurational) for the interacting residues of HigA2-HigB2 and CcdA-CcdB are shown as gray bars in the upper and bottom panels. Structures used for calculation are reported in Table S1. Residues with average frustration index above 0.78 (green line) are considered as minimally frustrated, residues bellow -1 (red line) are considered as highly frustrated residues. In between both borderlines, residues are neutral regarding frustration (Parra et al., 2016).


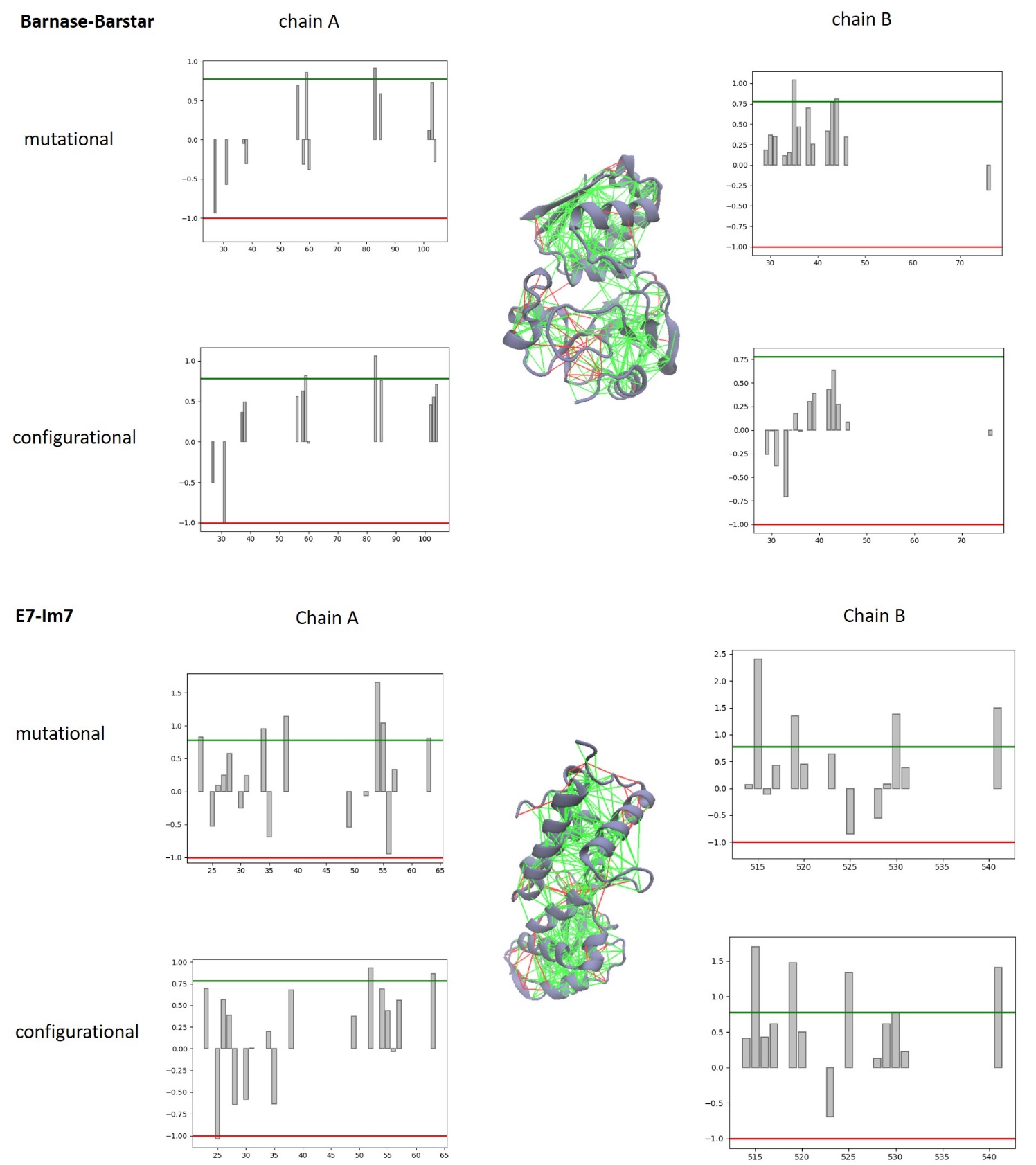


**Figure S6.** Frustration analysis of the globular-globular ultra-high affinity complexes. Frustration indices (mutational and configurational) for interacting residues of globular-globular complexes (barnase-barstar: upper panel, E7-Im7: bottom panel) are shown. Structures used for calculation are reported in Table S1. Residues with average frustration index above 0.78 (green line) are considered as minimally frustrated, residues bellow -1 (red line) are considered as highly frustrated residues. In between both borderlines, residues are neutral regarding frustration (Parra et al., 2016).


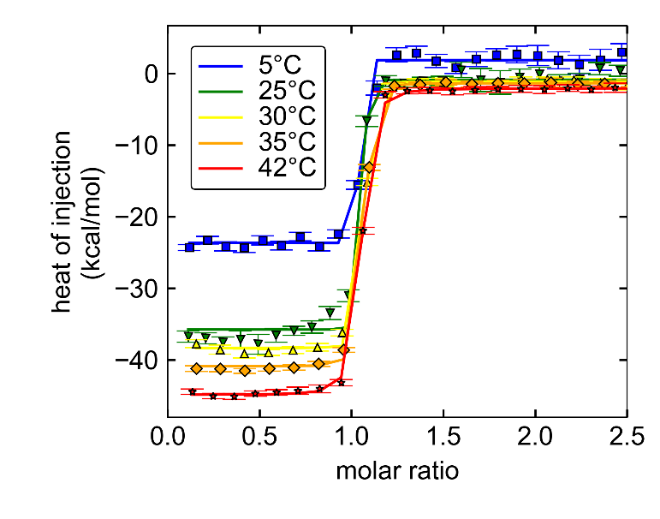


**Figure S7.** Determination of heat capacity change for HigA2_3-23_-HigB2 association. Full lines represent a global fit of the 1:1 binding model that assumes temperature-independent heat capacity change (Δ*C*_p_) associated with HigA2_3-23_-HigB2 association.

**References**

Drobnak, I., De Jonge, N., Haesaerts, S., Vesnaver, G., Loris, R., and Lah, J. (2013). Energetic Basis of Uncoupling Folding from Binding for an Intrinsically Disordered Protein. *J. Am. Chem. Soc.* 135, 1288–1294. doi:10.1021/ja305081b.

Frisch, C., Schreiber, G., Johnson, C. M., and Fersht, A. R. (1997). Thermodynamics of the interaction of barnase and barstar: Changes in free energy versus changes in enthalpy on mutation. *J. Mol. Biol.* 267, 696–706. doi:10.1006/jmbi.1997.0892.

Keeble, A. H., Kirkpatrick, N., Shimizu, S., and Kleanthous, C. (2006). Calorimetric dissection of colicin DNase-immunity protein complex specificity. *Biochemistry* 45, 3243–3254. doi:10.1021/bi052373o.

Parra, R. G., Schafer, N. P., Radusky, L. G., Tsai, M. Y., Guzovsky, A. B., Wolynes, P. G., et al. (2016). Protein Frustratometer 2: a tool to localize energetic frustration in protein molecules, now with electrostatics. *Nucleic Acids Res.* 44, W356–W360. doi:10.1093/nar/gkw304.

Wood, C. W., Ibarra, A. A., Bartlett, G. J., Wilson, A. J., Woolfson, D. N., and Sessions, R. B. (2020). BAlaS: fast, interactive and accessible computational alanine-scanning using BudeAlaScan. *Bioinformatics* 36, 2917–2919. doi:10.1093/BIOINFORMATICS/BTAA026.
